# Enhancement of dielectric and electric-field-induced polarization of bismuth fluoride nanoparticles within the layered structure of carbon nitride

**DOI:** 10.1038/s41598-020-71953-4

**Published:** 2020-09-09

**Authors:** Sarit K. Ghosh, Venkata K. Perla, Kaushik Mallick

**Affiliations:** Department of Chemical Sciences, University of Johannesburg, P.O. Box: 524, Auckland Park, 2006 South Africa

**Keywords:** Materials science, Nanoscience and technology

## Abstract

A single-pot, wet chemical method has been reported for the synthesis of bismuth fluoride nanoparticles (BF) and functionalized BF within the network of carbon nitride (BFCN). In BFCN, a structural transformation of BF, from cubic to pseudo-cubic (as evidenced by Rietveld refinement analysis), confirmed the contribution of carbon nitride (CN) on functionalization. The effect of functionalization of BF has been investigated through dielectric and field-induced polarization studies under different temperature and frequency conditions. Enhancement of dielectric constant values was noticed in BFCN as compared with BF system, in the order of 2.5 (30 °C) and 8.0 (100 °C) at 100 Hz. Fatigue-free maximum polarization values of 0.041 µC/cm^2^ and 0.054 µC/cm^2^, under the electric field of 5 kV/mm, were achieved for BF and BFCN samples, respectively, for 5 × 10^3^ cycles.

## Introduction

The pollution and non-sustainability are the major problems associated with fossil fuels, the main existing energy resource. The energy storage devices, those satisfy the requirement of higher capacity, faster recharge, environment friendly and lower cost, are in high demand. Several energy storage device concepts, such as, rechargeable batteries^1^, electrochemical supercapacitors^2,3^ and advanced dielectric capacitors^4^ have been recently developed. The dielectric materials with high energy density, minimum loss factor and wide range of temperature stability are the most favourite candidate for the energy storage capacitor application.

The use of low-cost and earth-abundant fluoride based materials have potential for the fabrication of energy storage devices. The fluorine atom has the highest electronegativity and small polarizability, which allows for the making strong and stable chemical bonds with other elements. Because of these properties, inorganic and organic fluorine compounds and fluoropolymers are employed for lithium batteries, fuel cells and capacitor applications^5^. Conversion-type fluorides have potential to achieve high energy density by involving the light and small fluoride anion^6^.

It has been reported that transition element based perovskite fluorides acted as potential electrode materials for rechargeable batteries^7,8^, where perovskite structure provide the robust architecture with intersectional tetragonal cavity chains^9^. Based on the above considerations, the bimetallic Co–Mn-based perovskite fluorides has been reported for the advanced electrode materials for supercapacitor application by exploiting the synergistic effect of Co and Mn redox species^10^. Though metal fluorides are insulator in nature and cannot be used as conventional electrode materials for device architecture, but at the lower voltage condition, microstrutured metal fluorides have been shown reversible reactivity^11^. It has been revealed that the nanostructured metal fluorides can facilitate the electrochemical performance when dispersed in a conductive matrix^12,13^, where the matrix contribute a high electrical conductivity and metal fluoride provide the electron tunneling path.

Recently, post-transition, non-toxic bismuth based materials attracted huge attention due to its ultrahigh energy storage performance^14,15^. However, very limited studies have been reported on bismuth fluoride related compounds. Efforts have been done to formulate the precise electrolytes that could enable the functionality of bismuth fluoride as positive electrodes while also maintaining stability at the negative electrode–electrolyte interface for the application in Li-ion battery^16^. The composite of bismuth fluoride and carbon black exhibit some interesting properties as an electrode material in supercapacitor application^17^. The luminescence^18^ and photocatalytic^19^ properties of bismuth fluoride based compounds have also been reported in the literature.

However, to the best of our knowledge, this is the first kind of report on the frequency dependent dielectric and field induced polarization performances of bismuth fluoride nanoparticles. A comparative investigation was also performed on the dielectric and polarization behaviour of functionalized bismuth fluoride under the influence of carbon nitride network. Both the materials exhibited a fatigue-free behaviour with excellent cycling stability during the electric field dependent polarization study. Carbon nitride has emerged as a promising material due to its facile synthesis route, thermal and chemical stability, and economic advantages. Carbon nitride exhibited encouraging performance in photocatalytic hydrogen evolution^20–22^, sensing^23^ and supercapacitor application^24^. Carbon nitride based materials also showed remarkable applications as a support system in organic transformation reaction^25–27^, organic photocatalytic reaction^28^, fuel cell^29^ and nonvolatile memory^30,31^ applications.

## Result and discussion

**Figure 1:**
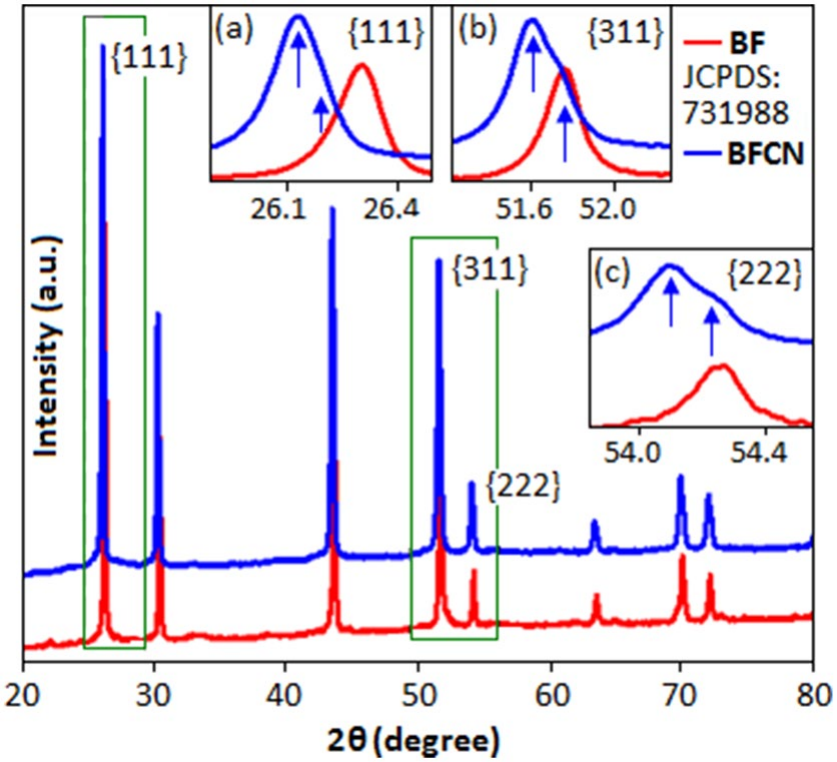
X-ray diffraction pattern of BF and BFCN within the range (2θ) from 20° to 80°. The peak shifting and splitting (blue arrow) for the preferred orientation of {111}, {311} and {222} planes is highlighted in the inset (a–c) respectively.

The Fig. 1 exhibits the X-ray diffraction pattern, measured by Philips PANalytical X'pert diffractometer, of BF and BFCN. All the diffraction peaks of BF and BFCN are matched according to cubic structure with the space group of Fm3m, JCPDS (Joint Committee on Powder Diffraction Standards) card number: 731988. The diffraction peaks indicate that the BF and BFCN materials are in single phase. The detailed peak profiles for the {111}, {311} and {222} planes are illustrated in the inset figures (a–c) respectively. The absence of splitting in {111}, {311} and {222} peaks revealed the perfect cubic structure of BF, whereas, in BFCN, the peak shifting and peak area broadening with the creation of shoulder-like appearance corresponds to cubic distortion of bismuth fluoride and formation of pseudo-cubic phase. The pseudo-cubic phase of bismuth fluoride in BFCN structure is due to the strong uniaxial stress, which was generated due to the lone pair-lone pair interaction between bismuth and nitrogen (from the CN). Concentration driven structural distortion and phase transition behaviour is usually associated with a peak splitting and broadening effects. These phenomenon has been reported to study the ferroelectric and electromechanical properties of bismuth based dielectric material^32,33^. In the present system the transformation from cubic to pseudo-cubic/rhombohedral structural is mainly observed in the region of {111, 311, 222} diffraction peaks. The mismatch of ionic radii of constituent elements may have significant impact on chemical heterogeneity that favours the structural distortion and create a cubic phase deficit region in BF_3_ for BFCN system^34,35^. Recently, phase change phenomenon was also reported in BiF_3_ nanoparticle system, where cubic to orthorhombic phase transformation was observed under the influence of pH^36^.

**Figure 2:**
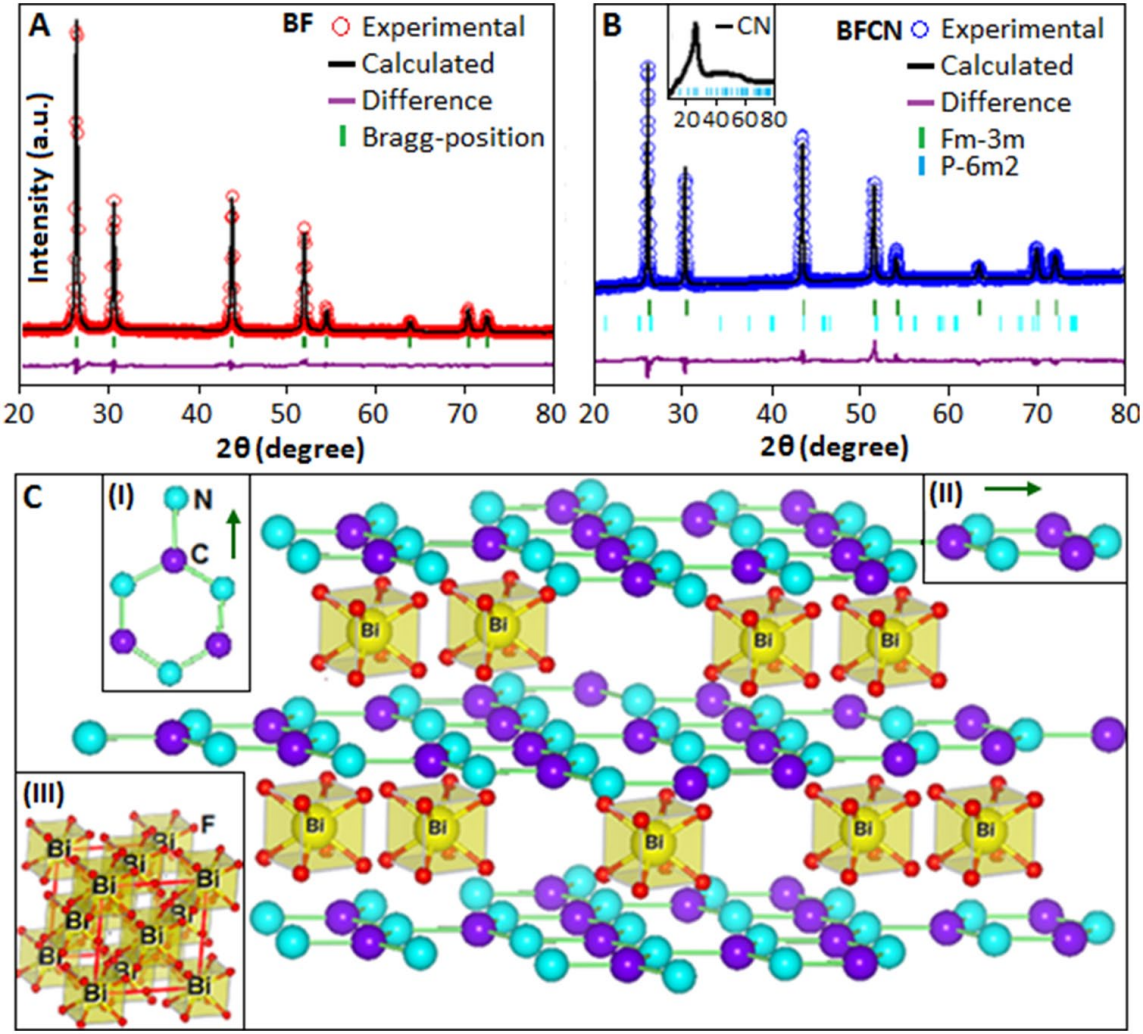
The experimental diffraction pattern of (**A**) BF (red) and (**B**) BFCN (blue), denoted by circle. The solid line (black) represents the calculated diffraction pattern. The difference between the curves denoted in pink colour and the vertical tick (green) indicates the Bragg’s position. The diffraction pattern of pure CN is shown in the inset (**B**). (**C**) Structural representation of BFCN. The unit cell representation of CN (inset: I, II) and BF (inset: III).

The Rietveld refinement analysis was performed for BF and BFCN using single phase cubic symmetry (Fm3m) and mixed phase of cubic (Fm3m) and hexagonal (P6m2) arrangement, respectively. The refined lattice parameters, atomic positions and the bond length of BF and BFCN materials are listed in the Table S1 (Supplementary Information). During the refinement analysis the occupancy factors were fixed to the nominal values and the convergent factor for BF (χ^2^ = 3.98, wR_p_ = 0.081 and R_p_ = 0.062) and BFCN (χ^2^ = 4.31, wR_p_ = 0.056 and R_p_ = 0.042) were restricted within the acceptable range. The sharp and well defined diffracted peaks for both BF (Fig. 2A) and BFCN (Fig. 2B) revealed the formation of crystalline bismuth trifluoride nanoparticles. The diffraction pattern of pure CN material is shown in the inset Fig. 2B and a prominent peak at 27.09° indexed to the graphitic plane of hexagonal packing structure (JCPDS: 871526). The magnified XRD figure of CN is available in the Supplementary Information, Fig. S1. The structure of CN consists of inter-planar staking of conjugated carbon–nitrogen rings. Among the different CN varieties, graphitic carbon nitride with hexagonal structure (P6m2) has been predicated energetically favourable structure^37–39^.

**Figure 3:**
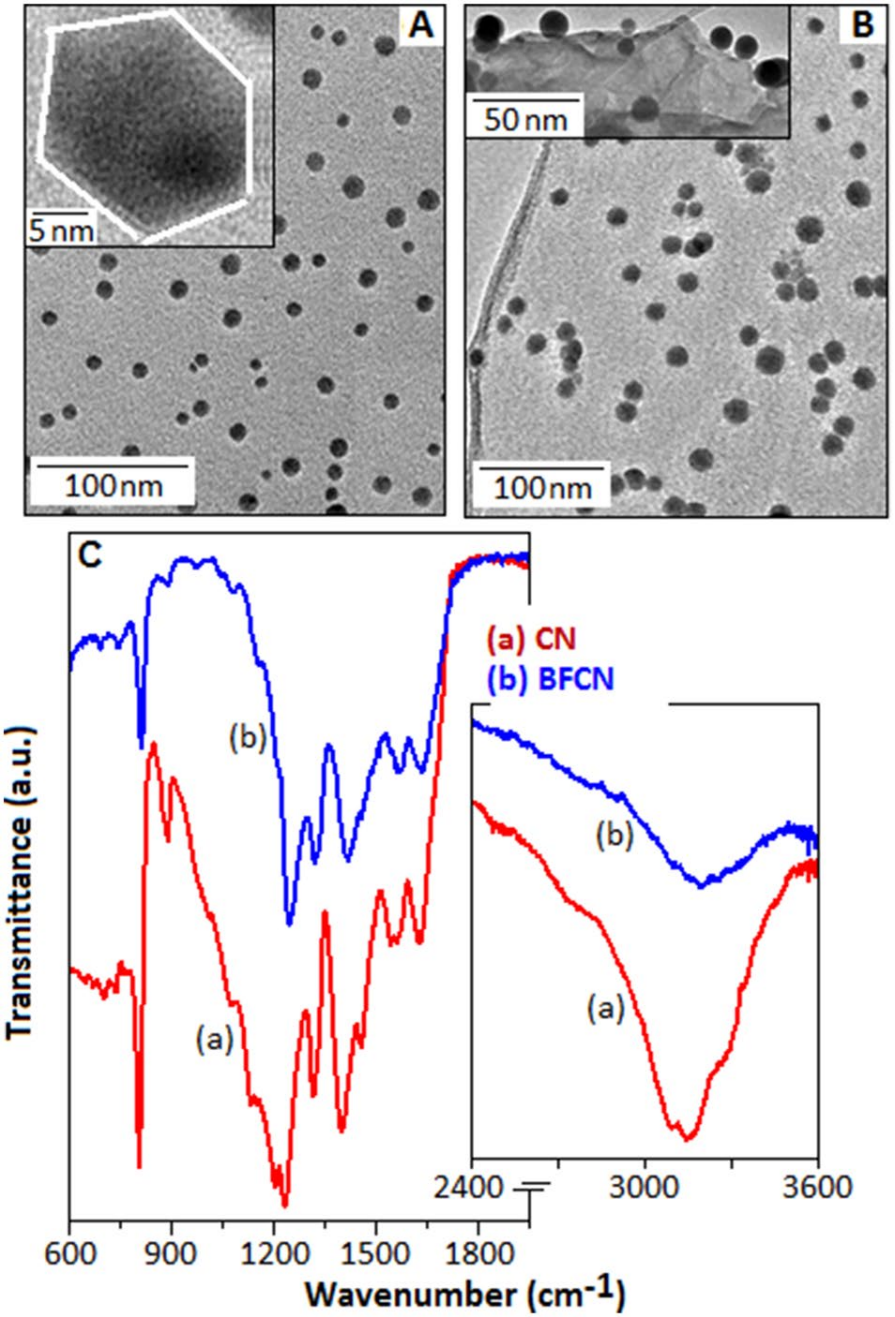
(**A**) TEM image of bismuth fluoride nanoparticles (inset: magnified image of a nanoparticle). (**B**) TEM image of bismuth fluoride nanoparticles in association with CN network (inset: bismuth fluoride particles located at the edge of the CN film and also within the layers of the films). (**C**) FTIR spectra of CN (a) and BFCN (b).

The schematic diagram, Fig. 2C, represents the functionalized and intercalated BF into the layered structure of CN. The layered structure of CN is constructed using the hexagonal symmetry parameters where, carbon and nitrogen atoms are interconnected in the planer of periodically arranged tri-s-triazine units. The vertical and horizontal view of a single triazine unit is illustrated in the inset (I) and (II), Fig. 2C, respectively. The unit cell of BF nanoparticles is constructed using the refine lattice parameters as shown inset (III). These structure consist of Bi-F polyhedra network centered at Bi cation (x, y, z = 0) formed a cage-like network^40^. Figure 3A displays the TEM image, JEOL (JEM-2100), of BF nanoparticles. The spherical shaped particles are seen with an average diameter of 16 nm, dispersed within the organic matrix. A high magnification TEM image is shown in Fig. 3A, inset. For the BFCN, the TEM image (Fig. 3B) indicate an enhancement of particle size. Figure 3B, inset, shows some of the bismuth fluoride particles located at the edge of the CN film and also within the layers of the films.

To identify the effect of BF on carbon nitride matrix, the infrared spectroscopic analysis was performed. The Fig. 3C shows the Fourier-transform infrared (FT-IR) spectra of (a) CN and (b) BFCN, which implies the existence of condensed aromatic carbon–nitrogen heterocycles. The IR-spectrum of the CN, Fig. 3C(a), displayed the vibrational bands at 1628 and 1556 cm^−1^, attributed to C=N stretching, while the bands at 1,227, 1,316, 1,401 and 1,456 cm^−1^ correspond to aromatic C–N stretching^41,42^. For the BiF_3_ functionalized carbon nitride sample (BFCN), the decrease of peak intensity and the shifting of the peak positions have been noticed, Fig. 3C(b), as compared with the CN sample. The bands responsible for C=N stretching vibration have been observed at 1634 and 1562 cm^−1^, whereas the aromatic C–N stretching vibration noticed at 1,243, 1,321 and 1,415 cm^−1^ for the BFCN sample. The triazine/*s*-triazine ring modes, correspond to condensed carbon–nitrogen heterocycles, detected at the wavenumber of 880 and 804 cm^−1^ for CN and 809 cm^−1^ for BFCN sample, with different intensities^43,44^. The vibration bands positioned at 1,456 and 880 cm^−1^ for the CN sample are not visible in the FTIR spectrum of BFCN sample. In the IR signal, the broad vibration bands within the spectral range of 3,000–3,500 cm^−1^ correspond to the N–H stretching and visible for both the samples but with different intencities^45^. By comparing the IR spectra of both CN and BFCN samples, it is evident that the IR peak positions for the BFCN sample are shifted towards the higher wavenumber values, as compared with CN sample, which denotes the decrease of bond length between carbon and nitrogen due to the change in electronegativity in association with the neighbouring atom. The above phenomenon indicates a successful functionalization of BF_3_ nanoparticles on the carbon nitride matrix.

**Figure 4:**
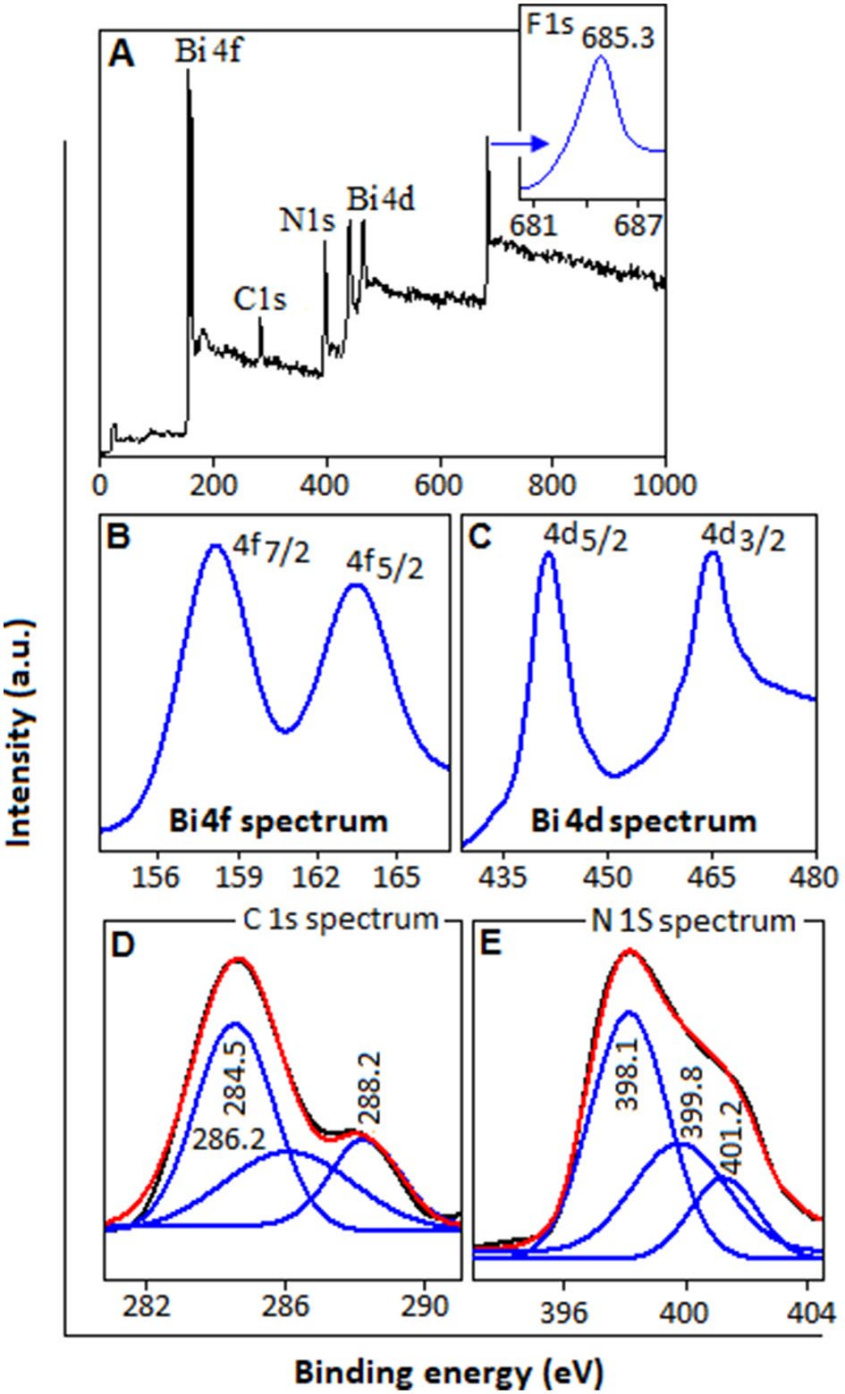
XPS spectra analysis of BFCN. (**A**) Survey spectrum, inset: F1s, (**B**,**C**) bismuth (4d and 4f.), (**D**) C1s and (**E**) N1s spectrum.

The chemical composition and chemical state of the as prepared BFCN has been analyzed by X-ray photoelectron spectroscopy (XPS) technique. Figure 4A displays the survey spectrum of the sample where XPS signals for carbon (1s), nitrogen (1s), bismuth (4d and 4f) and fluorine (1s) are distinctly visible. The high resolution F1s spectrum with the binding energy value of 685.1 eV (Fig. 4A, inset) indicate the existence of chemical environment of fluorine in the sample^46^. Two high intensity peaks with the binding energy values of 157.9 and 165.3 eV, correspond to Bi 4f_7/2_ and Bi 4f_5/2_, respectively^47^, Fig. 4B. The XPS peaks at 442.3 and 464.6 eV are assigned to Bi 4d_5/2_ and Bi 4d_3/2_, respectively^48^, Fig. 4C. Figure 4D shows the high resolution C1s spectrum of the BFCN system with the peak centered at 284.5 eV, which is ascribed to the C–C coordination in the sample^45,49,50^. The peak at 286.2 eV corresponds to sp^2^-bonded carbon in C=N, whereas the peak at 288.2 eV corresponds to sp^3^-bonded carbon in C–N of carbon nitride. The deconvolution of N 1s signal produced three peaks centered at 398.1, 399.8, and 401.2 eV, as shown in Fig. 4E. The peak at 398.1 eV is assigned to the sp^2^ C=N bond in the s-triazine ring, the peak at 399.8 and 401.2 eV, can be assigned to N atoms bonded with three C atoms^45,50^. The XPS analysis also confirmed the incorporation of BF in the carbon nitride network.

**Figure 5:**
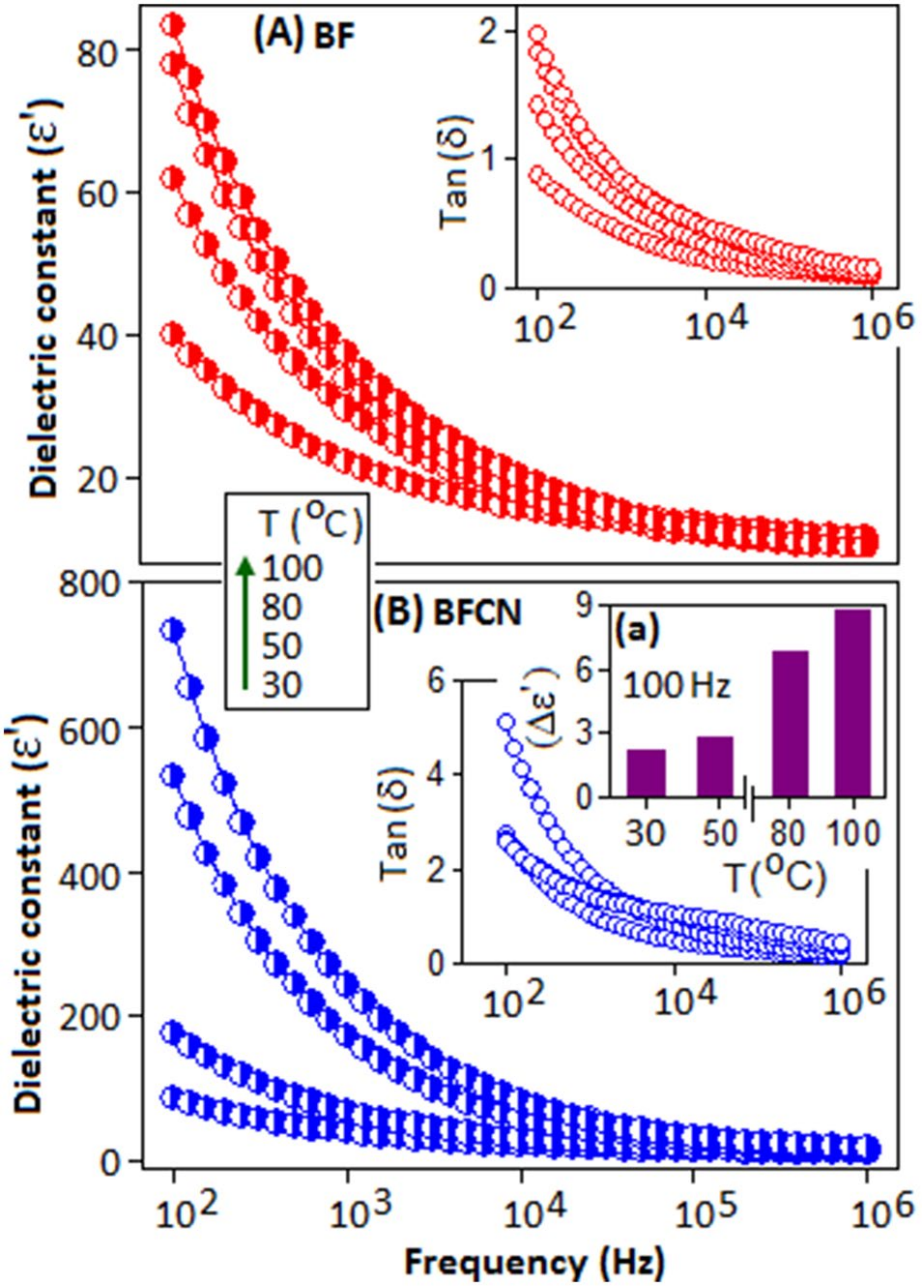
Frequency dependent dielectric constant (ε′) and dielectric loss factor (tanδ) (inset) of (**A**) BF and (**B**) BFCN. The increment of dielectric constant (Δεʹ) with temperature is illustrated in inset (a).

Figure 5A,B shows the frequency dependent dielectric constant (εʹ) for BF and BFCN, respectively, under four different temperature conditions (30, 50, 80 and 100 °C). The temperature and the frequency dependence dielectric studies were performed using HP 4284A LCR meter. The value of the dielectric constant is more pronounced at the low frequency region and decreases gradually with increase in frequency. At 30 °C, the dielectric constant values of BF and BFCN are 40 and 87, respectively, at 100 Hz. As the temperature increases, the dielectric constant values are increases for both the samples. At 100 °C a maximum value of 84 and 732 were achieved for BF and BFCN, respectively. The order of increment of dielectric constant ${(\Delta \varepsilon }^{{\prime}}={\varepsilon }_{BFCN}^{{\prime}}/{\varepsilon }_{BF}^{{\prime}}$) of BFCN, in comparison with BF, as a function of temperature is shown in Fig. 5B, inset (a). The higher dielectric constant values for BFCN could be explained by the Maxwell–Wagner (M–W) mechanism^51,52^, applicable for the inhomogeneous materials, where the incorporation of CN in BF offers an additional polarization to the system. Similar kind of effect has also been observed in other heterogeneous systems^53–55^. Towards lower frequency region the higher dielectric loss has been detected, Fig. 5A,B, in-set, due to the polarization loss for both the samples.

**Figure 6:**
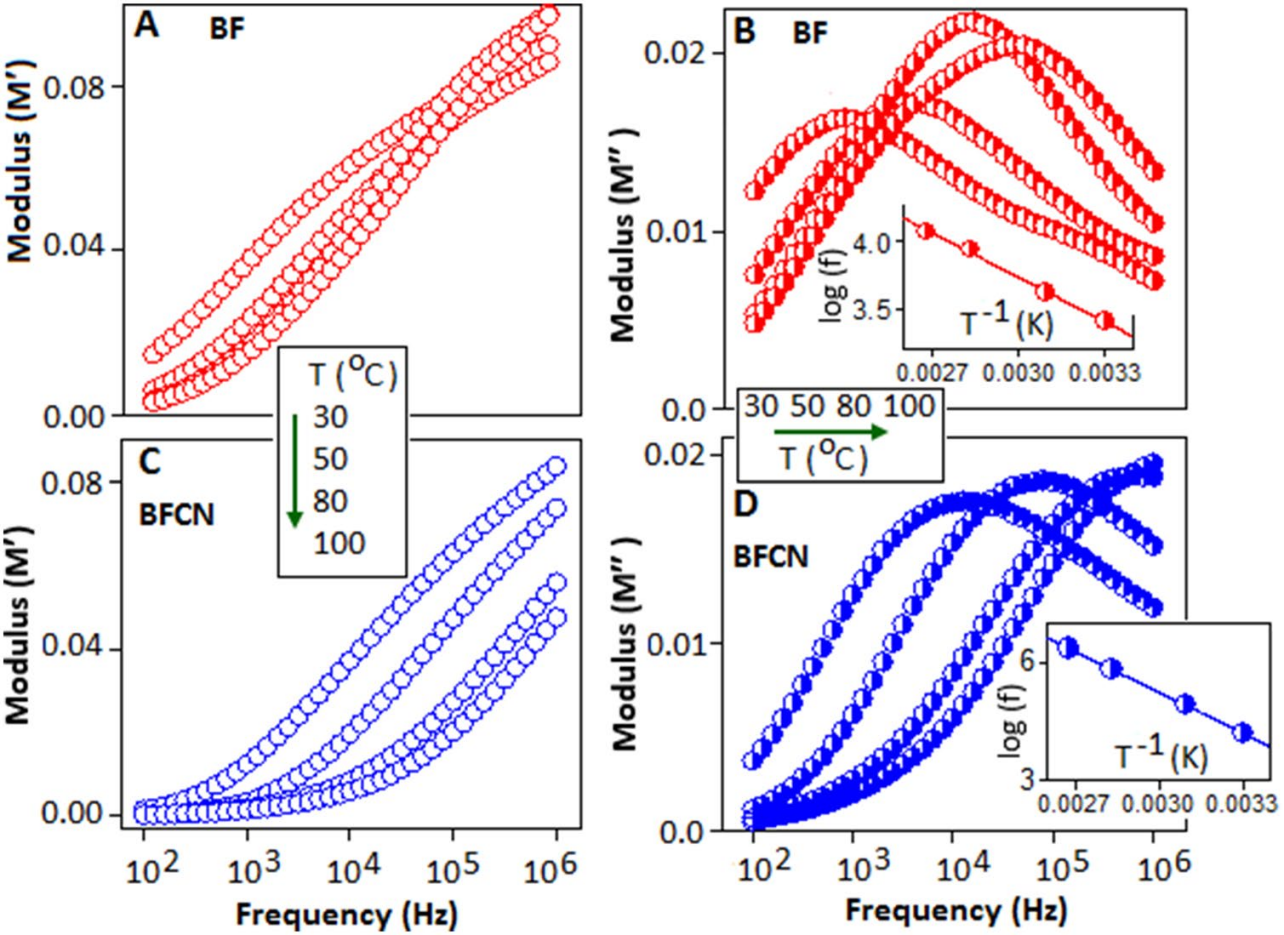
Variation of real (Mʹ) (**A**,**C**) and imaginary (Mʹʹ) (**B**,**D**) part of the electric modulus for BF and BFCN at different temperature. Arrow indicates the shifting of peak position with respect to the frequency.

Thermally activated relaxation mechanism is further illustrated in terms of dielectric modulus (M*) function, which is defined as,${M}^{*}= {M}^{{\prime}}+j{M}^{{{\prime\prime}}}=1/{\varepsilon }^{*}$ where, Mʹ and M″ are real and imaginary part of the electric modulus and ε* is the complex part of the dielectric constant. Figure 6A–D shows the real and imaginary part of the electric modulus as a function of frequency of BF and BFCN, respectively, measured under different temperature conditions (30, 50, 80 and 100 °C). The arrow indicates the direction of temperature. The value of Mʹ curves is very small towards the lower frequency regions, but increases with rising frequency for all temperatures. Distinct relaxation peaks are observed in the imaginary part (M″) of the modulus spectra for BF and BFCN, and shifted towards the higher frequency side with rising of temperature. The peaks in M″ spectra indicate the relaxation process with increasing temperature and exhibited a maximum value of M″_max_, centered at the dispersion region of Mʹ (*f*). The change in slope of M″ curve represents the mobility pattern of polarons from long range to short range with increasing frequency. The frequency below *f*_*max*_ the polarons are capable to tunnel over long distance, whereas the frequency above *f*_*max*_ the polarons are confined in potential well and travel in short distance. The shifting and broadening effect of the peaks are more prominent with increasing of temperature, which indicate the distribution of relaxation time, an evidence of non-Debye type of relaxation mechanism. The peak maxima shifting towards higher frequency direction with increase in temperature indicate the carrier motion become faster and thermally activated^56^. The peak position, *f*_m_, is plotted as a function of T^−1^, using Arrhenius relation,${f}_{m}= {f}_{0}exp\left(-{E}_{a}/{K}_{B}T\right)$, where, *f*_0_ is the pre-exponential factor, *E*_*a*_ is the activation energy of the relaxation. The calculated activation energy values for BF and BFCN are 0.23 eV and 0.25 eV and corresponding *f*_0_ values are 16.3 and 39.1 Hz, respectively. A higher value of activation energy of BFCN indicate that the trapped polarons of BF nanoparticles within the CN layer need more energy to overcome the potential barrier for the relaxation process. Thus trapped polarons of BFCN is likely to participate in the polarization mechanism to enhance the dielectric constant value. At higher temperature and applied field, the polarons are thermally activated and hopped to the nearest lattice sites and generate the polaronic relaxation within the material.

**Figure 7:**
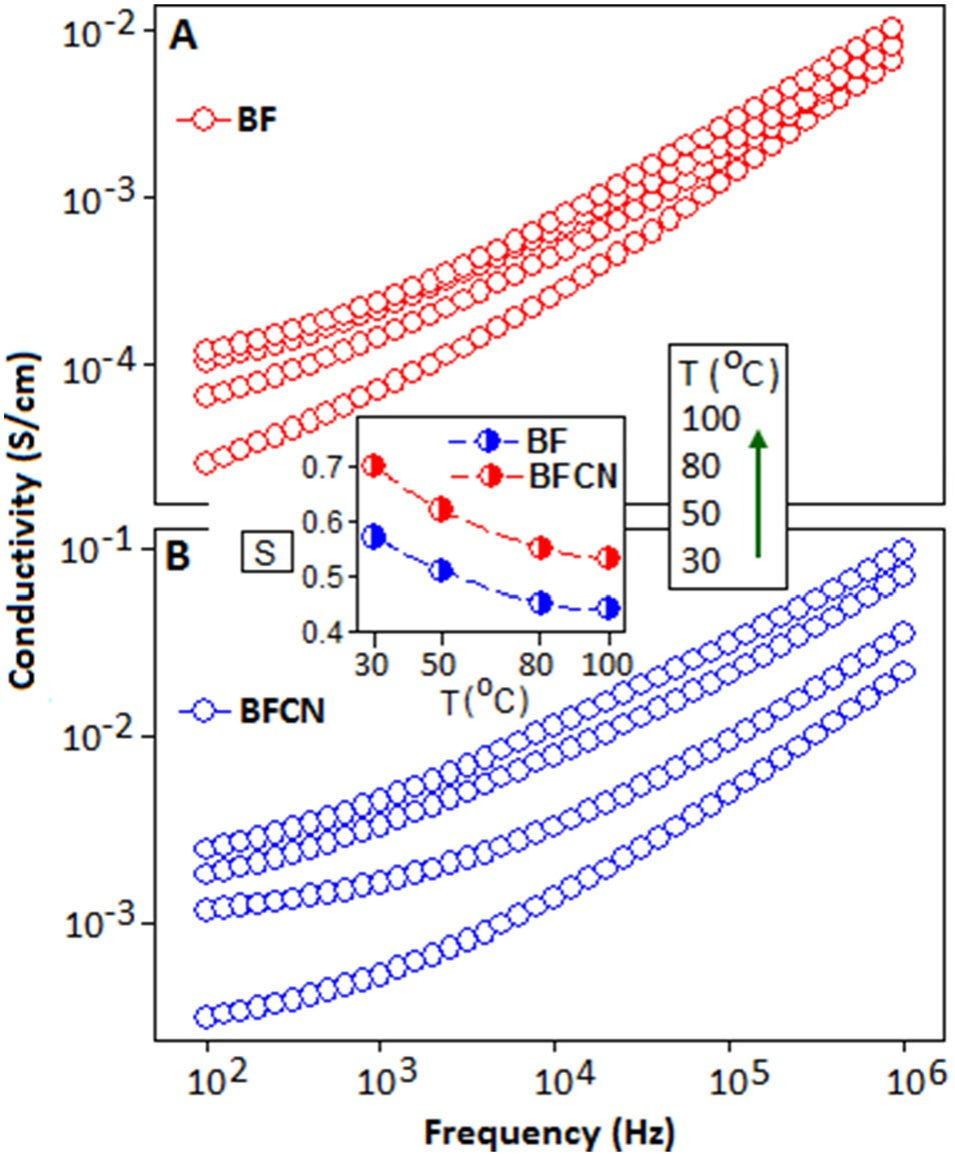
AC conductivity on a log–log scale of (**A**) BF and (**B**) BFCN at different temperatures (30, 50, 80 and 100 °C) and the arrow indicates the raising in temperature. Inset shows the variation of parameter ‘s’ as a function of temperature.

To understand the polaronic movement in BF and BFCN, the AC-conductivity of both the materials were measured under different temperature condition and the results are exhibited in the Fig. 7A,B, respectively. From the figure it is cleared that the AC-conductivity (*σ*_*AC*_) increases with increasing temperature and frequency conditions. In the figure, at lower frequency region a weak plateau-like feature is observed, usually referred to the DC-part of the conductivity. The overall AC-conductivity is represented by the universal power law relation, ${\sigma }_{AC}= {\sigma }_{DC}+ A(T){\omega }^{S}$, where, ${\sigma }_{DC}$ is the DC-part of the conductivity, A is the temperature dependent pre-factor and ‘s’ is the frequency and temperature dependent parameter varies within the range of 0 < *s* < 1, which predict the type of conduction mechanism. The parameter ‘s’ extracted from the linear fitting of the power relation and the results are plotted in Fig. 7 (inset). The value of ‘s’ for BF and BFCN decreases with increasing temperature, which implies that the polaron hopping mechanism took place between the potential barrier^57,58^. We have found that with the increase of temperature the value of hopping barrier energy (W_H_) decrease and at the higher temperature small polaron tunneling is privileged due to higher internal energy of the particles (electronic Supplementary Information). The hopping distance (*R*_*H*_) is calculated from correlated barrier-hopping (CBH) model. The extracted *R*_*H*_ values are directly proportional with temperature (Table S2, Supplementary Information), which may be attributed to the long range polaron hopping phenomena. All the extracted values (s, W_H_ and *R*_*H*_) are listed in Table S2 (Supplementary Information). The trends of *R*_*H*_ indicate that the polaron hopping took place between the inter-polyhedra unit of BF via the organic matrix. However, the *R*_*H*_ value for BFCN is small compare to the BF, which is probably due to the layer structure of CN that further contribute the shorter hopping distance within the material. Similar behaviour has been reported in other organic–inorganic hybrid materials, where polaron movement is dominated along the inter-planner axis^59^.

**Figure 8:**
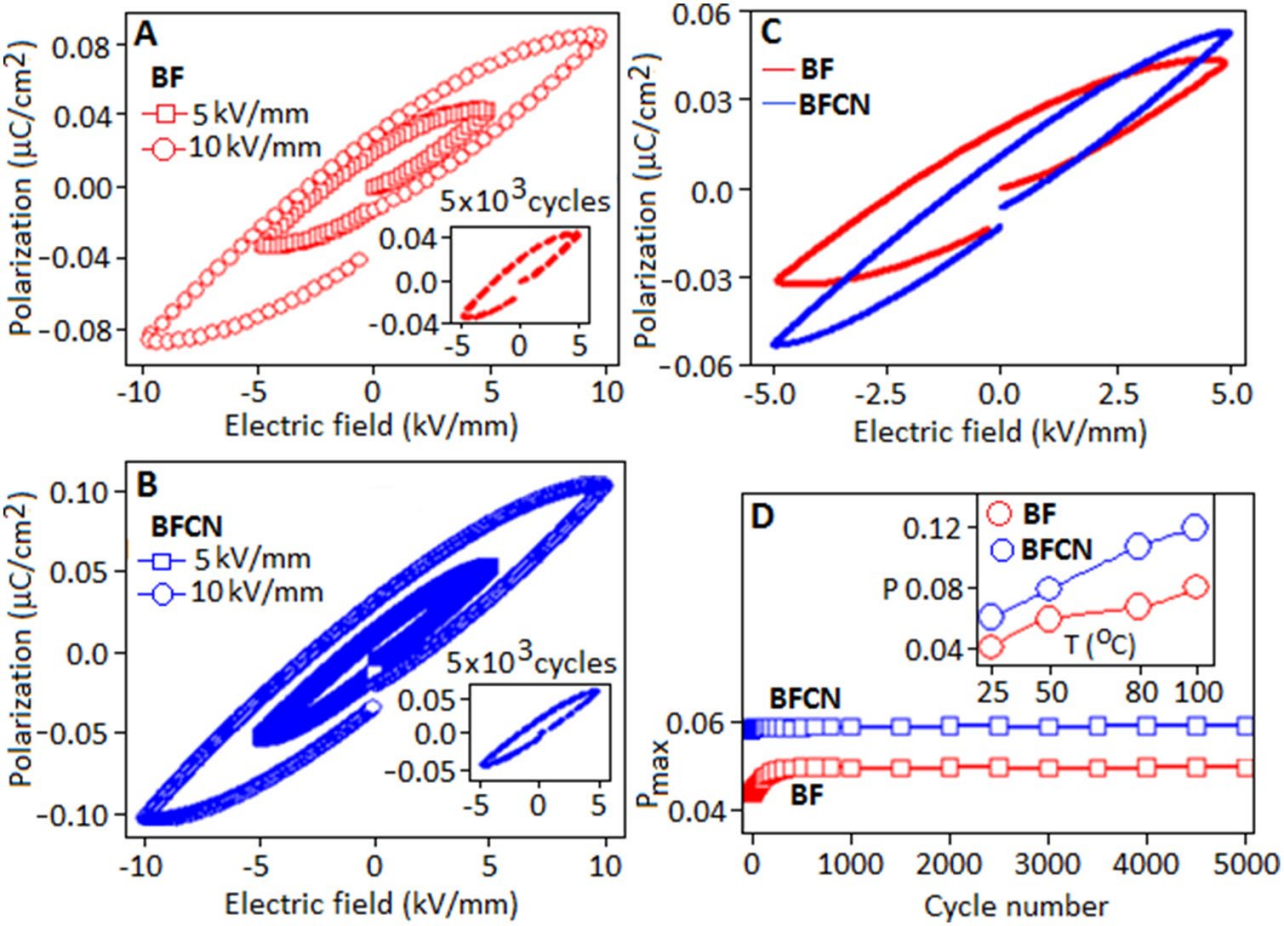
Polarization versus electric field (P-E) hysteresis loop of (**A**) BF and (**B**) BFCN. The inset shows the behaviour of P-E loop for a period of 5 × 10^3^ cycles. (**C**) Comparison of P-E loops for BF and BFCN materials, (**D**) maximum polarization (P_max_) for 5 × 10^3^ cycles. The change in polarization value with respect to temperature (inset).

The polarization versus electric field (P-E) hysteresis loop was investigated to determine the charge separation and storage properties of the materials. Figure 8A,B show the room temperature hysteresis loop of BF and BFCN, respectively, measured at 5 kV/mm and 10 kV/mm (electric field conditions). P-E loops were measured by using triangular wave with a frequency of 100 Hz. At room temperature, the maximum polarization (P_max_) values are achieved for BF and BFCN materials 0.041 µC/cm^2^ and 0.054 µC/cm^2^, respectively under 5 kV/mm, as shown in Fig. 8C. The enhancement of polarization value in BFCN is possibly due to the interface coupling effect from the CN matrix^60^. It is important to mention that the difference in the electric properties between the constituent components would promote the conduction and the polarization process, simultaneously. The incorporation of carbon nitride in bismuth fluoride generate local electric field at the interfaces, which subsequently promotes the polarization response within the material^61–63^. In the P-E loop for BFCN, a slight shift has been noticed towards negative electric field direction which is presumably originated due to the presence of asymmetric environments. It is also important to mention that in the present case a huge discrepancies has been found between the calculated^64,65^ and experimental value of the polarization, possibly due to the centrosymmetric (cubic) and non-ferroelectric nature of the materials. The BF and BFCN were subjected to switching for 5 × 10^3^ cycles to evaluate the effect of electric field dependent polarization stability. Both the materials exhibited a fatigue-free characteristic without any degradation, Fig. 8A,B: inset, with the consistent values of maximum polarization and remanent polarization during the cycling process. The maximum polarization as a function of cycle number exhibited a fatigue free behaviour for both the samples (Fig. 8D). The variation of P-E loop pattern with temperature is displayed in Fig. 8D (inset).

**Figure 9:**
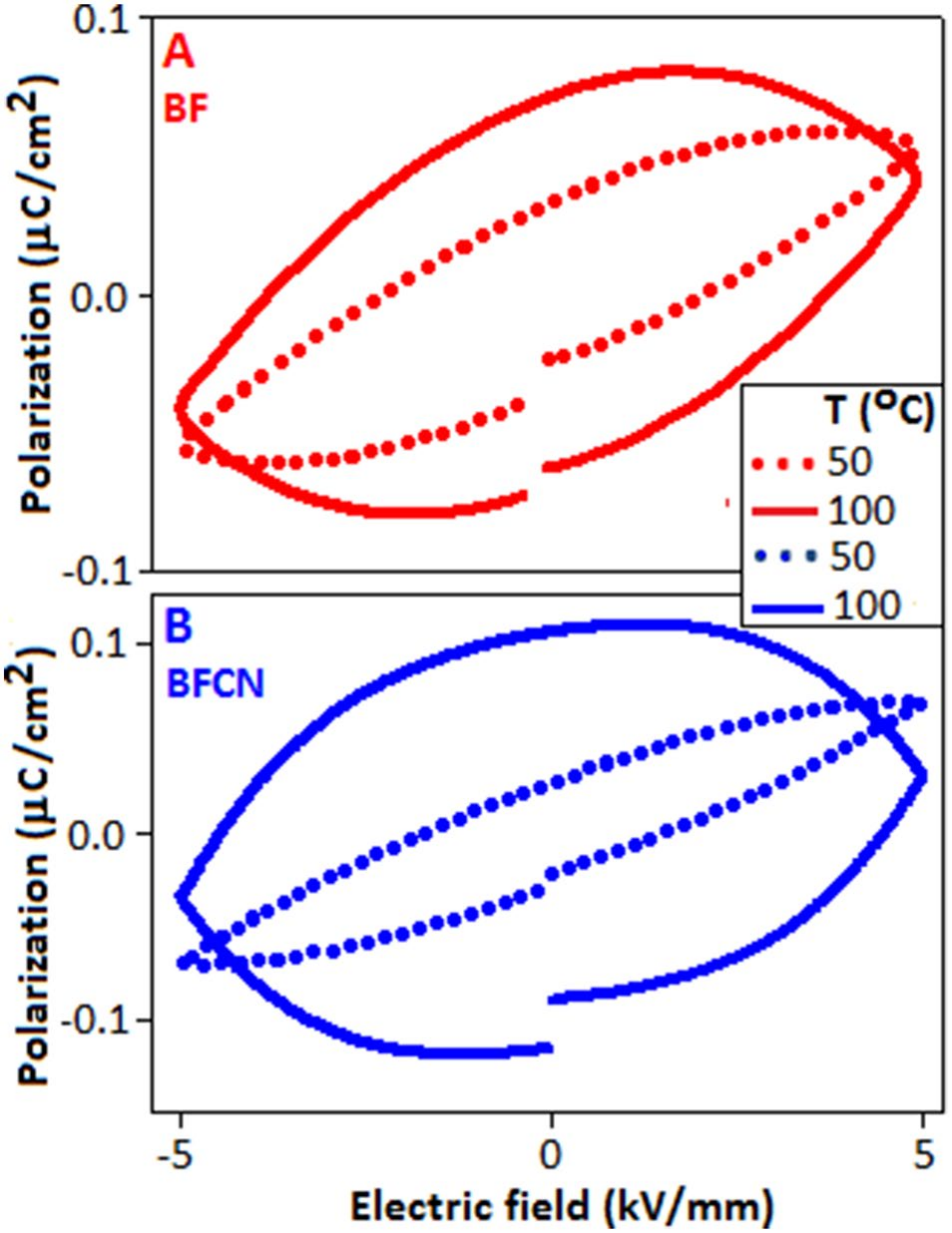
Temperature dependent polarization hysteresis for (**A**) BF and (**B**) BFCN.

The maximum polarization for BF and BFCN are obtained 0.082 µC/cm^2^ and 0.124 µC/cm^2^, respectively, at 100 °C, when measured under the electric field of 5 kV/mm, Fig. 9A,B, respectively. The larger polarization loop area of BFCN, as compared with BF, indicates higher charge accumulation in the BFCN due to the interfacial polarization, as already mentioned before. Similar behaviour has been reported in other nano composite systems, where conducting nano fillers are responsible for the enhancement of polarization^66^.

## Conclusion

Superior dielectric and polarization performances has been reported due to the integration of carbon nitride in bismuth fluoride, where functionalization took place between bismuth and nitrogen through lone pair-lone pair interaction with an intercalated network. A successful intercalation resulted a structural transformation of bismuth fluoride and also enhanced the surface polarization effect. Our results projected that polaron hopping mechanism was followed in the frequency dependent conduction process for both bismuth fluoride and functionalized bismuth fluoride within carbon nitride network. In BFCN, a larger charge accumulation at the interfaces causes a higher conduction process, which also facilitate an enhancement of polarization and dielectric performance. To the best of our knowledge, this is the first kind of report on the polarization and dielectric property of functionalized bismuth fluoride based nanomaterial that could have a potential in low dimensional hybrid capacitor application. A literature review illustrates (Supplementary Information, Table S3) the previously reported bismuth fluoride based materials and their potential applications.

## Experimental section

### Synthesis of bismuth fluoride nanoparticles (BF) and bismuth fluoride–carbon nitride (BFCN) nanocomposite

#### BF nanoparticles

BF nanoparticles were synthesized using a single-pot, wet chemical method under ambient temperature condition^17^. In a typical reaction, 6 ml of aqueous solution of bismuth nitrate (0.16 M) was drop-wise added to 1.5 ml of aniline (diluted in 10 ml methanol) under constant starring condition in a conical flask. A white precipitation was immediately formed at the bottom of the flask. To this precipitation, freshly prepared ammonium fluoride solution (5 ml of 0.1 M) was added slowly under starring condition and left the material for 6 h under nitrogen atmosphere.

#### BFCN nanocomposite

The graphitic phase of carbon nitride (CN) was synthesized using a previously reported method^67^. For the synthesis of BFCN nanocomposite system, 5 wt% CN was dispersed in methanolic solution of aniline and a similar procedure was followed as above.

Both the precipitation were collected using filtration method and dried at 80 °C under vacuum in an oven for 2 h.

## Supplementary information

Supplementary Information
