# Supplementary material for: Enhancement of dielectric and electric-field-induced polarization of bismuth fluoride nanoparticles within the layered structure of carbon nitride
Source: Sci Rep. 2020 Sep 9;10:14835. doi: 10.1038/s41598-020-71953-4 (PMC7481291; doi:10.1038/s41598-020-71953-4)
Supplement: Supplementary file 1 — Supplementary Information [file 41598_2020_71953_MOESM1_ESM.docx]

**Supplementary information**

Enhancement of dielectric and electric-field-induced polarization of BiF_3_ nanoparticles within the layered structure of carbon nitride

Sarit K Ghosh, Venkata Perla, Kaushik Mallick

Department of Chemical Sciences, University of Johannesburg, P.O. Box: 524, Auckland Park, 2006, South Africa.

The hopping barrier energy (W_H_) of the polaron was calculated from the following relation:

$s=1-\frac{6K_{B}T}{W_{H}-K_{B}T ln\left( 1/{\omega\tau_{0}} \right)}$ (1)

Where, *K_B_* is the Boltzmann constant,

T is the temperature and

*τ_o_* is the relaxation time of the polaron carriers.

As, *W_H_* >> *K_B_T ln* (1/ωτ_0_), so the relation can be rewritten as,

$s=1-\frac{6K_{B}T}{W_{H}}$ (2)

The calculated values of *W_H_* are listed in the table S2.

The hopping distance (*R_H_*) was calculated from the following relation: $R_{H}= \frac{e^{2}}{\pi\varepsilon\varepsilon_{0}\left[ W_{H}-K_{B}T ln\left( 1/{\omega\tau_{0}} \right) \right]}$ (3)

Where, e is the electronic charge, *ε* is the dielectric constant of the material, *ε_0_* is the dielectric constant of the free space.

**Table: S1**

**
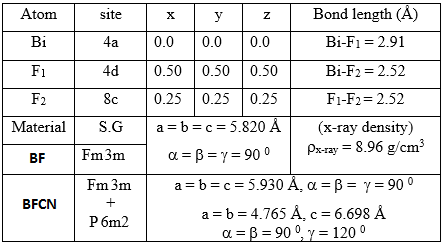
**

**Table S1:** Refined lattice parameters, atomic positions, x-ray density and bond length of BF and the lattice parameters of BFCN in the mixed phase symmetry.

**Table: S2**


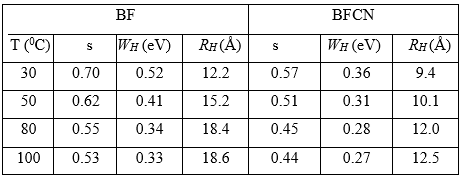


**Table S2:** Values of the extracted parameters (s), hopping barrier potential (*W_H_*) and hopping distance (*R_H_*) of BF and BFCN materials at different temperature condition.

**Table: S3**

| Materials | Synthesis | Morphologies | Application and property study | Ref. |
| --- | --- | --- | --- | --- |
| BiF_3_ (NC) | Solvent extraction | Rod, plate and wire shaped | - | R1 |
| BiF_3,_ Eu-BiF_3_,  and BiOF (NP) | Homogeneous precipitation | Spherical  and octahedra | Luminescence | 18 |
| BiF_3_ (NP) | Water bath | Agglomerated | Photo-catalysis,  band structure | 19  R2 |
| BiF_3_ (NC) | Colloidal | Hexagonal | Electro-chemical | R3 |
| BiF_3_ (NP) | Wet chemical | Spherical | Supercapacitor | 17 |
| BiF_3_ (NP)  BiF_3_-CN (NP)  (reported first time) | Wet chemical | Spherical | (a) Rietveld refinement,  (b) Dielectric constant,  (c) Polarization and fatigue study | In this  work |

**Table S3:** Comparative analysis of previous reported BiF_3_ nanoparticles and the present studies of BF and BFCN materials synthesized at similar laboratory condition. (NC = nanocrystal, NP = nanoparticle)


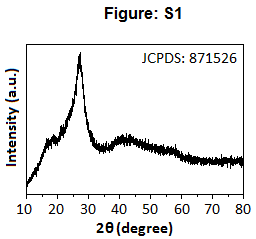


**Figure S1:** The diffraction pattern of pure CN.

References

R1. Zhao, J. M., Pan, H. L., He, X., Wang, Y. S., Gu, L., Hu, Y. S., Chen, L. Q., Liu, H. Z. & Dai, S. Size-controlled synthesis and morphology evolution of bismuth trifluoride nanocrystals via a novel solvent extraction route. *Nanoscale* **5**, 518-522, (2013).

R2.   Zhang, W., Yin, J. R., Wu, W. H., Xie, W., Zhang, P. & Ding, Y. H. Study on structure and properties of transition metal doped bif3 by first-principles. *Physica E* **80**, 125-129, (2016).

R3. Oszajca, M. F., Kravchyk, K. V., Walter, M., Krieg, F., Bodnarchuk, M. I. & Kovalenko, M. V. Colloidal bif3 nanocrystals: A bottom-up approach to conversion-type li-ion cathodes. *Nanoscale* **7**, 16601-16605, (2015).
